# Supplementary material for: Almond By-Products Substrates as Sustainable Amendments for Green Bean Cultivation
Source: Plants (Basel). 2024 Feb 16;13(4):540. doi: 10.3390/plants13040540 (PMC10891745; doi:10.3390/plants13040540)
Supplement: Supplementary file 1 [file plants-13-00540-s001.zip › plants-2857652-supplementary.pdf]

Table S1. Pearson Correlation matrix between biochemical parameters of pods resulting from green beans grown in four distinct substrates, irrigated at 100% and 50% of WFC.

**Variables**	Chl a	Chl b	Total Chl	Chl (a/b)	Total Carot.	Total (Chl/Carot.)	Phenolics	*Ortho*-diphenols	Flavonoids	AC-DPPH	AC-ABTS	Proteins
Chl a	1											
Chl b	**0.456***	1										
Total Chl	**0.916****	**0.776****	1									
Chl (a/b)	**0.885****	-0.008	**0.624****	1								
Total Carot.	-0.031	-0.040	-0.040	-0.017	1							
Total (Chl/Carot.)	0.241	0.271	0.294	0.138	**-.930****	1						
Phenolics	-0.100	0.224	0.030	-0.234	0.015	0.113	1					
Ortho-diphenols	0.044	**0.425***	0.223	-0.168	**0.411***	-0.255	**0.577****	1				
Flavonoids	-0.231	0.183	-0.082	-0.333	**0.420***	-0.278	**0.427***	**0.714****	1			
AC-DPPH	-0.212	0.214	-0.053	-0.319	0.367	-0.241	**0.426***	**0.582****	**0.732****	1		
AC-ABTS	-0.234	-0.216	-0.263	-0.155	**0.805****	**-.895****	-0.191	0.155	0.146	0.249	1	
Proteins	0.200	**0.532****	0.382	-0.057	0.336	-0.176	**0.549****	**0.835****	**0.566****	**0.582****	0.144	1

*. The correlation is significant at the 0.05 level (2 ends).

**. The correlation is significant at the 0.01 level (2 ends).

Bold values indicate very strong and strong correlations between variables. Abbreviations: Chl a – chlorophyll *a*, Chl b – chlorophyll *b*, Chl (a/b) – chlorophyll *(a/b)* ratio, Total Chl – total chlorophyll *(a+b*), Total Carot. – total carotenoids, Total (Chl/Carot.) – total (Chl/Carot.) ratio, AC-ABTS – antioxidant activity by ABTS method, AC-DHHP – antioxidant activity by DPPH method.
